# Michigan appropriateness guide for intravenous catheters in pediatrics — miniMAGIC-Brasil: translation into Brazilian portuguese

**DOI:** 10.1590/1984-0462/2024/42/2023159

**Published:** 2024-05-13

**Authors:** Marcelle Di Angelis Ambar Felipe, Maria Angelica Sorgini Peterlini, Amanda Ullman, Mavilde da Luz Gonçalves Pedreira

**Affiliations:** a Universidade Federal de São Paulo, São Paulo, SP, Brazil.; b University of Queensland, Brisbane, UQ, Austrália.

**Keywords:** Infusions, intravenous, Nursing pediatrics, Vascular access devices, Pediatrics, Patient safety, Infusões intravenosas, Enfermagem pediátrica, Dispositivos de acesso vascular, Pediatria, Segurança do paciente

## Abstract

**Objective:**

To perform the translation and adaptation of the Michigan Appropriateness Guide for Intravenous Catheters in Pediatrics (miniMAGIC) into Brazilian Portuguese.

**Methods:**

Methodological study performed in five recommended stages: initial translations; synthesis of the translations; back translations; assessment of the back translations; expert committee assessment. The expert committee was composed of three registered nurses and two doctors who had a Master’s and/or PhD degree, and an expertise in intravenous therapy and pediatric and neonatal care. To assess the semantic, idiomatic, experiential and conceptual adequacy, a Likert scale was applied, in which 1, “not equivalent”; 2, “inequivalent”; 3, “cannot assess”; 4, “quite equivalent”; 5, “totally equivalent”. The terms mostly analyzed as negative in equivalence and with a lower than 20 score were reviewed and submitted to a new assessment, with the Delphi Technique until consensus was obtained. The results were stored in electronic spreadsheets and treated with concordance index, with a minimum acceptable result of 0.80.

**Results:**

The content of all recommendations, named as miniMAGIC-Brasil, was validated by the expert committee after two stages of evaluation. All recommendations had an overall agreement index of 0.91.

**Conclusions:**

The miniMAGIC-Brazil guide was validated in respect to the adequacy of the translation after two steps.

## RESUMO

**Objetivo:**

Realizar a tradução e adaptação do miniMAGIC para a língua portuguesa do Brasil.

**Métodos:**

Estudo metodológico realizado em cinco etapas: traduções iniciais; síntese das traduções; retrotraduções; avaliação das retrotraduções; avaliação por comitê de especialistas. O comitê foi composto de três enfermeiros e dois médicos que possuíssem pós-graduação *stricto sensu* e experiência em acesso vascular e pediatria. Para a análise de adequação semântica, idiomática, experiencial e conceitual de cada item do instrumento, foi empregada escala tipo Likert graduada em 1, como “não equivalente”; 2, “pouco equivalente”; 3, “não sei avaliar”; 4, “bastante equivalente”; e 5, “totalmente equivalente”. Os termos majoritariamente analisados como negativos quanto à equivalência e com pontuação inferior a 20 foram revisados e submetidos a nova análise segundo a técnica Delphi até que o consenso fosse alcançado. Os resultados obtidos foram armazenados em planilhas eletrônicas e tratados com análise do índice de concordância mínimo de 0,80.

**Resultados:**

O conteúdo de todas as recomendações, que receberam nomeação de miniMAGIC-Brasil, alcançou índice de concordância mínimo, segundo julgamento por comitê de especialistas, após duas etapas de avaliação. Todas as recomendações obtiveram índice de concordância ao final de 0,91.

**Conclusões:**

O guia miniMAGIC-Brasil obteve índice de concordância dos termos segundo análise da adequação da tradução após duas etapas.

## INTRODUCTION

To ensure efficacy, effectiveness, and safety in intravenous (IV) therapy it is necessary to choose the best vascular access device (VAD) to meet the clinical, therapeutic, and personal needs of the patient and family.^1^ Choosing the most appropriate VAD in pediatrics can be especially complex, with the need to consider a wide range of clinical and anatomical factors.^2^

More than half of hospitalized children have one indication for peripheral venipuncture, and at least one in three require new punctures before completing the proposed treatment.^3,4^ Failure to obtain venous access brings discomfort and anxiety to the child and family, being reported by parents as one of the most negative experiences of hospitalization.^5^ Furthermore, they reduce the efficiency of the treatment, compromise the child’s vascular health, increasing recovery time, length of hospital stay and costs for the institution, as they demand a greater amount of material and time from the health professional.^6^

There are many guidelines available to support evidence-based decision-making surrounding VAD device selection in pediatrics.^7^ This decision is dynamic, complex and, due to the large number of available guidelines, there may be conflict in clinical decisions of the multidisciplinary team, exposing the patient to possible risks.^8^

Building upon the Michigan Appropriateness Guide for Intravenous Catheters (MAGIC), the Michigan Appropriateness Guide for Intravenous Catheters for Pediatrics (miniMAGIC) was published in 2020, following the RAND/University of California at Los Angeles — UCLA Appropriateness method.^9-11^ The RAND/UCLA appropriateness method includes systematic reviews, followed by case scenarios adjudicated by an expert panel, who report on an intervention’s appropriateness, balancing risk with benefit. The miniMAGIC recommendations were divided into seven populations: hospitalized full-term newborns, hospitalized infants, hospitalized children and/or adolescents, stable and unstable critically ill patients, children with congenital heart diseases, and with dependence on long-term vascular devices, without parenteral nutrition.^10^ In addition to the populations, the recommendations are structured according to the IV indication and predicted length.^12^ The recommendations are based on levels of scientific evidence, being classified as appropriate, uncertain, inappropriate and disagreement.

In order to enable the use of miniMAGIC in Brazil, this study aimed to translate the miniMAGIC content into Brazilian Portuguese. In the future, miniMAGIC-Brasil can be used to support the clinical decision of the team of physicians and nurses in the choice of VADs, promoting evidence-based care and access, for Brazilian healthcare professionals, to a relevant instrument for pediatrics.

## METHOD

Methodological study designed to translate miniMAGIC from English to Portuguese spoken in Brazil, based on the consensus-based standards for the selection of health measurement instruments (COSMIN) recommendations.^13^

This study included the following steps: initial translations, synthesis of translations, back-translations, evaluation of back-translations and content validation by a committee of experts.^14,15^

The main author of miniMAGIC (AJU) consented to the translation of the original instrument, as well as collaborated with the study team. The study was approved by the Research Ethics Committee of the Federal University of São Paulo under Certificate of Presentation for Ethical Appreciation (CAAE) registration number 50075521.4.0000.5505.

The study sample was composed by the expert committee, chosen by convenience, targeting nurses and physicians specializing in pediatric and neonatal care, who met the inclusion criteria. It was expected to be a five participants group.

It included professionals with an academic degree at Master’s and/or PhD level, advanced knowledge of the English language, experience in VAD and pediatric or neonatal care, additional expertise in the field of pediatric or neonatal VAD (evidenced by published papers and/or presentations at specialist/scientific meetings).

A search to identify the inclusion criteria and eligible experts was carried out on the Lattes Platform of the National Council for Scientific and Technological Development (CNPq), using the search limiter “subject” and Brazilian nationality. The Medical Subject Headings (MeSH) terms used in the search were: intravenous therapy, pediatric intensive care, neonatal care, pediatric surgery, pediatric nursing and vascular access device.

The initial online search in the curriculum also included academic degree, level of English proficiency, publications of papers in international journals, presentations at specialist/scientific national and international meetings, being part of a research group related to the study theme and actual activity in academy or clinical practice.

This strategy resulted in six possible experts, three nurses and three physicians. The invitation was sent by email to five possible participants, as the sample was limited, including an Informed Consent Form. Of the five specialists initially selected, one did not answer the invitation, and the sixth participant was approached and informed consent approval was obtained.

Thus, the expert panel was multidisciplinary, composed by two physicians and three nurses who endorsed their knowledge and experience with the study subject, and all of them were part of consolidated departments or research group in VAD, pediatric intensive care and patient safety.

The process of translating and adapting miniMAGIC followed the order of initial translations from English into Portuguese, synthesis of the translated content, back-translations from Portuguese into English, evaluation of the back-translations by the author of the original study, and validation of the content translated into Portuguese by the expert panel.

Initially, miniMAGIC was translated into Brazilian Portuguese by two independent professionals, whose native language was Portuguese.^13^ The first professional (P1) selected had expertise with the content of the study and advanced knowledge of the English language.^14^ The second (P2) was not familiar with the subject, having been hired due to his advanced knowledge of the English language and recognized competence in translation activity.^15^

After producing the two translated reports, P1 and P2, the synthesis stage (S1) was carried out, in which a specialist in IV therapy and pediatrics evaluated the translated documents and prepared a single report.^13^

Subsequently, a meeting was held over the Internet between this professional and the group of researchers to reach consensus on differences in translations or suggestions for adaptation from a clinical perspective.

With proposal S1 done, the back-translation was carried out into English, the original language of miniMAGIC. This step was carried out by two hired professional translators, who had English as their native language and no knowledge about the content of the study.^13^

In this process, participants worked independently and produced reports entitled English 1 (E1) and English 2 (E2).

Once the back-translation process was finished, miniMAGIC was analyzed by the author of the original study. She analyzed the back-translated guides and, after a meeting to discuss the results, she gave her agreement with the produced documents.

After the original author’s approval, the expert panel assessment was carried out using the Delphi Technique. The panel was instructed to evaluate all terms from four perspectives of adaptation into the Portuguese language: that of semantic equivalence, for evaluation of the translation; the idiomatic perspective, with an analysis of the idiomatic expressions of the original version in the translation; the experiential perspective, verifying whether the questions of clinical routine were culturally applicable, and the conceptual perspective to check if the terms had different meanings according to the cultural aspect.

Each term presented could be evaluated as “not equivalent”, “less equivalent”, “I don’t know how to evaluate”, “fairly equivalent” and “fully equivalent”. The first classification listed was scored as 1 and the last as 5.

Data collection was developed in two phases. In the first, from July 25 to November 15, 2021, translation of the original miniMAGIC was carried out, producing the translation, synthesis and back-translation documents. It was recommended that the expert committee only validate the synthesis document. The other versions produced were available for the committee to consult, if necessary, as well as the original published miniMAGIC.^10^

The second phase was developed from December 7, 2021, to May 11, 2022, where the validation of the terms by the expert committee was held. The synthesis document was transcribed into an electronic form and divided into the sections “Validation for vascular access devices terminologies”, with the presentation of all terms referring to vascular devices, comparatively, between the English and Portuguese languages, and “Validation of the miniMAGIC-Brasil guides and summary of recommendations”.

For each question there was a space left for comments or suggestions regarding the proposed translation, if the specialist deemed it necessary. Only terms with a minimum score of 20, without the classification of “not equivalent”, were considered validated. Those that were not validated, with a score of less than 20 and/or with an indication of “not equivalent”, were submitted to reassessment in the second round using the Delphi Technique, until consensus was reached by the committee. The forms were prepared electronically on the Google Forms^®^ platform.

Data were imported from the Google Forms^®^ platform and stored in a Microsoft Excel^®^ spreadsheet, protected with a personal password for access. The results were obtained after the sum of the scores for each evaluated item, according to the assessment of each expert. For each item, the minimum value could be 5 and the maximum 25.

The content validity index (CVI) and the content validity ratio (CRV) were calculated for each item and for each miniMAGIC-Brasil recommendation. The CVI aimed to evaluate the level of agreement between the judges, considering the scores 4 and 5.^16^ The minimum CVI for validation was previously settled at 0.80.^16,17^ The RVC was calculated as a way to validate the translated terms, being considered as adequate the result that reached 1.00.^18^ To assess the internal consistency of each recommendation guide, alpha Cronbach’s coefficient was used with a previously settled minimum value of 0.70.^19^

## RESULTS

The results obtained from the two rounds of the miniMAGIC translation assessment are presented next. As a decision of the original author, the name of the instrument miniMAGIC would not be translated. The Brazilian research team, as agreed with the original author, proposed that the translated name would be miniMAGIC-Brasil.

The global analysis of the results expressed in Table 1 showed that the terminologies reached a CVI greater than 0.80, being validated in the first round of the Delphi Technique with an acceptable agreement between the evaluators, RVC 1.00. The validated term “*Intraóssea* — IO” received a suggestion to be changed into “*Intraósseo* — IO”, being revised and submitted to the expert panel for a second round of the Delphi Technique. The suggestion was not deemed relevant and the term “*Intraóssea* — IO” was kept.

**Table 1.** Translation and adaptation into Brazilian Portuguese of the Vascular Access Devices terminologies identified in the miniMAGIC instrument, carried out by the expert panel in the first and second rounds of the Delphi Technique.

Vascular access devices terminologies translation	Score (min. – max.)	CVI	RVC
First round			
Umbilical	25 (5)	1.00	1.00
Cateter Intravenoso Periférico — CIP	24 (4–5)	1.00	1.00
Cateter Intravenoso De Linha Média — CILM	22 (4–5)	1.00	1.00
Cateter Intravenoso Central De Inserção Periférica — PICC	23 (4–5)	1.00	1.00
Cateter Intravenoso Central Não Tunelizado — CICNT	22 (4–5)	1.00	1.00
Cateter Intravenoso Central Tunelizado Com *Cuff* — CICTc	21 (4–5)	1.00	1.00
Cateter Intravenoso Central Tunelizado Sem *Cuff* — CICTs/c	21 (4–5)	1.00	1.00
Cateter Intravenoso Central Totalmente Implantado — CICTI	23 (4–5)	1.00	1.00
Intraóssea — IO*	24 (4–5)	1.00	1.00
Second round			
Intraóssea — IO	25 (5)	1.00	1.00

min.: minimum value; max: maximum value; CVI: content validity index; RVC: content validity ratio.

* terms with suggestions made by the expert panel.

The term “*Cateter Intravenoso Central de Inserção Periférica*” (PICC) had its acronym kept from the English term PICC after being considered usual in the synthesis stage. As for the different group ages and characteristics of children included on miniMAGIC, all terms were validated. For “*Lactentes Hospitalizados*”, an item with low agreement between the panel, the CVI was 0.84 and received a suggestion to change be changed into “*Lactentes Hospitalizados (*≤ *1 ano)*”. After the second round of the Delphi Technique, it reached a CVI of 1.00 (Table 2).

**Table 2.** Translation and adaptation into Brazilian Portuguese of the age groups and characteristics of the children identified in the miniMAGIC instrument, carried out by the expert panel in the first and second rounds of the Delphi Technique.

Terms translation	Score (min. – max.)	CVI	RVC
First round			
População: Recém-nascidos a termo hospitalizados	24 (4–5)	1.00	1.00
População: Lactentes hospitalizados*	21 (2–5)	0.84	0.60
População: Crianças e/ou adolescentes hospitalizados*	24 (4–5)	1.00	1.00
População: Paciente gravemente enfermo — Estável	22 (4–5)	1.00	1.00
População: Paciente gravemente enfermo — Instável	23 (4–5)	1.00	1.00
População: Fisiologia univentricular*	20 (3–5)	0.80	0.60
População: Circulação biventricular	24 (4–5)	1.00	1.00
População: Longa permanência, sem NP	23 (4–5)	1.00	1.00
Idade: Recém-nascido	25 (5)	1.00	1.00
Idade: Lactente*	23 (4–5)	1.00	1.00
Idade: Criança e/ou adolescente*	24 (4–5)	1.00	1.00
Second round			
População: Lactentes hospitalizados (≤ 1 ano)	22 (4–5)	1.00	1.00
População: Crianças (>1 ano) e/ou adolescentes hospitalizados	24 (4–5)	1.00	1.00
População: Fisiologia funcionalmente univentricular	22 (4–5)	1.00	1.00
Idade: Lactente (≤ 1 ano)	23 (4–5)	1.00	1.00
Idade: Crianças (>1 ano) e/ou adolescentes hospitalizados	24 (4–5)	1.00	1.00

min.: minimum value; max: maximum value; CVI: content validity index; RVC: content validity ratio.

* terms with suggestions made by the expert panel.

The same occurred for “*Infante*” and “*Criança e/ou Adolescentes*” which received suggestions for changing into “*Infante (*≤ *1 ano)*” and “*Criança (>1 ano) e/ou Adolescentes*”. Although the term “*Fisiologia univentricular*” reached a CVI of 0.80, it received suggestions to be changed into “*Fisiologia funcionalmente univentricular*”, being submitted for the second round and reaching a CVI of 1.00.

Of all the 36 terms that composed the guides and summary of recommendations, only one (2.7%) did not reach the level of agreement in the first round of the Delphi Technique, which was “*Dispositivo adequado*”. “*Infusão compatível com acesso periférico*”, “*Infusão não compatível com acesso periférico*”, “*Adequação do dispositivo*”, “*Adequação do dispositivo: Superior, superior do corpo*” e “*Adequação do dispositivo: Inferior, inferior do corpo*” received sugestions to be changed and were submitted for the second round of the Delphi Technique, thus reaching a maximum CVI of 1.00 (Table 3).

**Table 3.** Translation and adaptation into Brazilian Portuguese of the terms identified in the miniMAGIC instrument, carried out by the expert panel in the first and second rounds of the Delphi Technique.

Terms translation	Score (min. – max.)	CVI	RVC
First round			
Adequado	21 (2–5)	0.84	0.60
Incerto	25 (5)	1.00	1.00
Inadequado	24 (4–5)	1.00	1.00
Indicação: Compatível com infusão periférica*	20 (2–5)	0.80	0.60
Indicação: Incompatível com infusão periférica*	21 (2–5)	0.84	0.60
Indicação: Coleta frequente de sangue (mais de uma vez ao dia)	23 (4–5)	1.00	1.00
Duração: ≤ 7 dias	25 (5)	1.00	1.00
Duração: 8 - 14 dias	25 (5)	1.00	1.00
Duração: ≥31 dias	25 (5)	1.00	1.00
Dispositivo adequado^†^	18 (2–5)	0.60	0.20
G- Gauge	22 (3–5)	0.88	0.60
F- French	23 (4–5)	1.00	1.00
Monitorização hemodinâmica	25 (55)	1.00	1.00
Curta duração (<15 dias)	24 (4–5)	1.00	1.00
Longa duração (≥15 dias)	24 (4–5)	1.00	1.00
Estágio: Estágio 1	24 (4–5)	1.00	1.00
Estágio: Estágio 2	24 (4–5)	1.00	1.00
Estágio: Estágio 3	24 (4–5)	1.00	1.00
Dispositivo adequado: Superior*	19 (2–5)	0.80	0.60
Dispositivo adequado: Inferior*	19 (2–5)	0.80	0.60
Jugular	23 (4–5)	1.00	1.00
Subclávia	23 (4–5)	1.00	1.00
Femoral	23 (4–5)	1.00	1.00
Indicação: Uso contínuo	22 (4–5)	1.00	1.00
Indicação: Uso intermitente (ao menos uma vez ao dia)	21 (4–5)	1.00	1.00
Dispositivo	23 (4–5)	1.00	1.00
Indicação clínica: Sem dificuldade ou urgência	20 (4–5)	0.80	0.60
Indicação clínica: Difícil	24 (4–5)	1.00	1.00
Indicação clínica: Difícil	24 (4–5)	1.00	1.00
Indicação clínica: Urgente	24 (4–5)	1.00	1.00
Indicação clínica: Não urgente	23 (4–5)	1.00	1.00
Adequação: Antebraço, mão, pé, couro cabeludo, fossa cubital	22 (4–5)	1.00	1.00
Veias: Basílica, braquial, cefálica, safena magna, axilar, femoral na porção mediana da coxa, jugular interna			
Inserção: Na fossa cubital	23 (4–5)	1.00	1.00
Inserção: Acima da fossa cubital	24 (4–5)	1.00	1.00
Second round			
Indicação: Infusão compatível em acesso periférico	23 (4–5)	1.00	1.00
Indicação: Infusão não compatível em acesso periférico	23 (4–5)	1.00	1.00
Adequação do dispositivo	22 (4–5)	1.00	1.00
Adequação do dispositivo: Superior, superior do corpo	20 (4)	1.00	1.00
Adequação do dispositivo: Inferior, inferior do corpo	20 (4)	1.00	1.00

min.: minimum value; max: maximum value; CVI: content validity index; RVC: content validity ratio.

* terms with suggestions made by the expert panel;

† not validated terms.

The individual miniMAGIC parts have descriptions and subtitles, highlighting that all terms reached a minimum level of agreement in the first round of evaluation. Recommendation 7 received a suggestion to change the term “*locais de inserção*” to “*sítios de inserção*”, which was approved by the expert panel, after the second round of the Delphi Technique.

In the description of Recommendation 1, one of the specialists mentioned the abbreviations of the devices, which was not observed in the original version. In discussion with the research team, it was decided to keep the descriptions of the abbreviations in order to better understanding and avoid misinterpretations on clinical practice.

All miniMAGIC-Brasil recommendations obtained an average CVI of 0.91. The Cronbach’s alpha calculations were: Recommendation 1 (0.642), Recommendation 2 (0.717), Recommendation 3 (0.493), Recommendation 4 (0.752), Recommendation 5 (0.481), Recommendation 6 (0.536), Recommendation 7 (0.707). Recommendations 1, 3, 5 and 6 showed lower levels of internal consistence.

**Figure 1.**
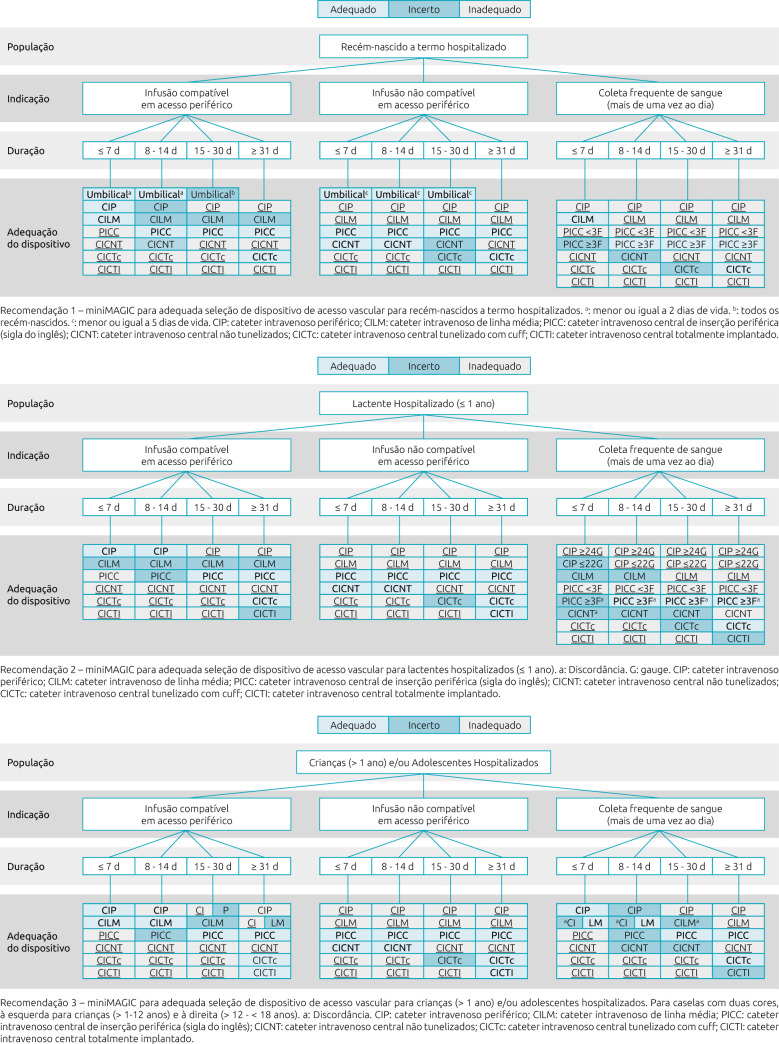
Translated version of miniMAGIC-Brasil — Recommendations 1, 2 and 3.

The complete instrument is shown from Figure 1 to Figure 3. In the description of each recommendation the term “disagreement” appears. The original publication emphasizes the need for further clinical investigation and scientific evidence due to divergences in assessment between the panel of experts.

**Figure 2.**
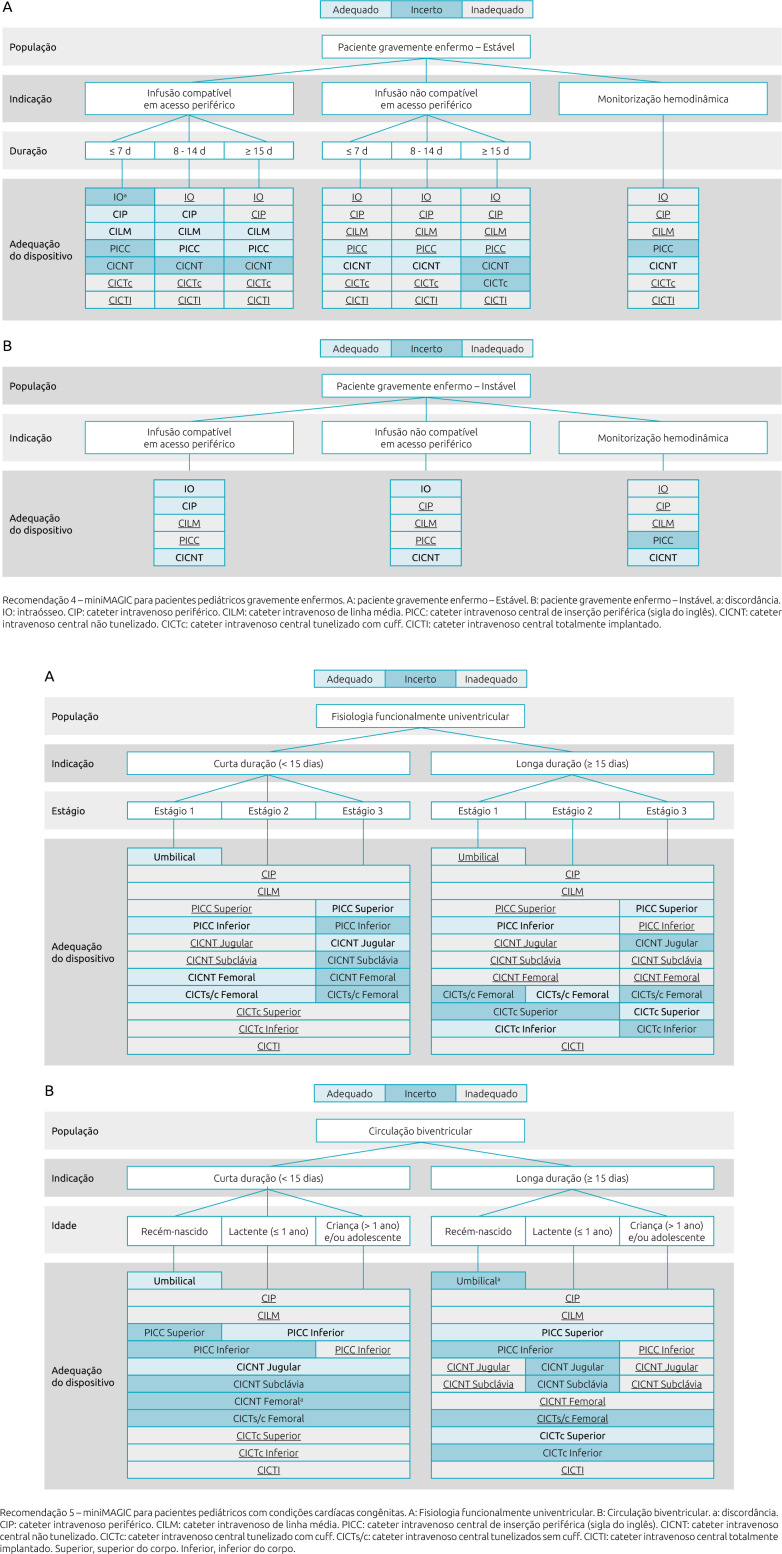
Translated version of miniMAGIC-Brasil — Recommendations 4 and 5.

**Figure 3.**
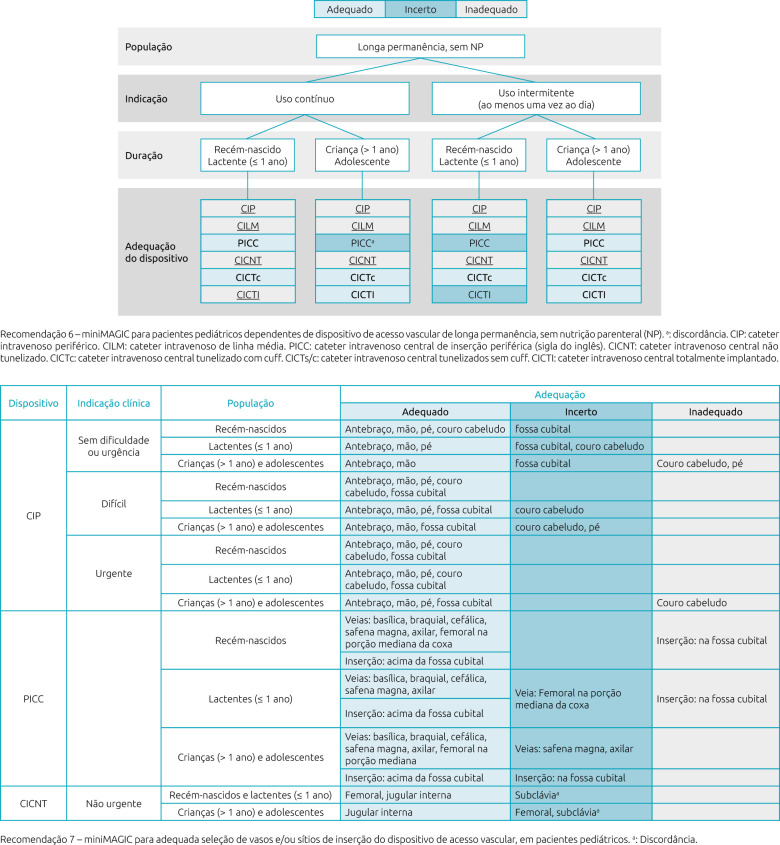
Translated version of miniMAGIC-Brasil — Recommendations 6 and 7.

## DISCUSSION

The methodological process of translation into Portuguese proposed for the miniMAGIC was successfully validated after following a high rigorous approach, reaching consensus among specialists in two rounds of the Delphi Technique, with an overall CVI of 0.91. Despite the complexity of the instrument, the consensus was reached in two rounds of evaluation.

The translation assessment by the expert panel compared the final version obtained from the synthesis document with the original version, as the other documents had already been judged by the research group, reaching a high level CVI when compared to other studies. When analyzing translation and cultural adaptation studies published about pediatric care, the CVI reached were more than 0.90, like miniMAGIC-Brasil, which demonstrates an assertive approach.^20,21^

Beyond the CVI, in this study, the Cronbach Alfa coefficient was calculated in order to evaluate the internal consistence of each miniMAGIC recommendation. When analyzing the results, the coefficient was acceptable in three recommendations, which shows a good assessment without the transcultural adaptation, which is still in progress, when compared to other studies.^22,23^

The great consensus obtained in this study can be attributed to a number of factors. Specifically, the consolidated group of researchers in the area of pediatric vascular access, participation of the main author of the study in the research and selection of professionals in the field of pediatrics with advanced clinical and academic experience in vascular access.

Each recommendation provides guidance regarding the best appropriateness of the VAD, based on the child’s developmental characteristics and clinical and therapeutic needs, being classified by the colors: green for adequate, red for inadequate and yellow for uncertain. This can alert the professional in clinical practice as to the risky choices for the success of the intervention and patient safety.

The potential for miniMAGIC-Brasil to influence Brazilian healthcare practice is extensive. The misuse of a VAD can cause serious injuries to patient. A prospective observational study involving children admitted in Brazilian hospitals (n=19) found that 63% had their IV therapy interrupted because of complications such as infiltration, dislodgment and phlebitis. Each of these children also required a new VAD inserted.^24^ Peripheral intravenous catheter-associated complications are a worldwide phenomenon, so strategies to reduce this harm need to similarly global.^4^

Central venous catheters, such as PICCs, are at high risk of significant complications such as bloodstream infections and thrombosis.^25,26^ Judicious selection of these devices, in comparison to peripheral devices, is a core recommendation within miniMAGIC and miniMAGIC-Brasil. In a descriptive study conducted with nurses and nurse technicians who worked in neonatal and pediatric, it was asked which VAD was selected for the patient and the results showed that, regardless of the age group and duration of the IV therapy, the CIP was the only device used.^27^ The use of miniMAGIC-Brasil can provide additional information to clinicians and can potentially result in a more varied and appropriate approach to vascular access in children and newborns, thereby reducing infections and thromboses.

The use miniMAGIC-Brasil in clinical practice can also provide a strategy to promote better communication among nurses and physicians. It can support professionals’ evidenced-based decision making, promote the process of systematization of care, and contribute to the creation of VAD care bundles, improving its correct use, optimizing the patient’s treatment and preserving vascular health.^28^ This harmonization will hopefully reduce interdisciplinary confusion, and improve clinical outcomes for patients, optimizing therapy with reduced length of stay, and reducing unnecessary costs.^29^

The translation process carried out in this study opens up possibilities for the implementation of miniMAGIC-Brasil across the country, with a subsequent clinical research application to assess the influence of this tool in clinical practice. As future steps, miniMAGIC-Brasil will be incorporated into an information and communication technology application to facilitate its use.

Therefore, the miniMAGIC guide was successfully translated into Brazilian Portuguese, being titled miniMAGIC-Brasil and reaching high levels of internal consistency level (0.70–0.90). Its development has some limitations, since it is not yet finished and the instrument is not as yet ready for clinical application. This is because low levels of RVC were observed in Recommendations 1, 3, 5, and 6, requiring a transcultural adaptation for proper understanding, which is in progress in the main research. Only after this is done can the instrument be safely used in Brazil’s healthcare system, aiming to reduce vascular access-associated harm in children.

## Data Availability

The database that originated the article is available with the corresponding author.
